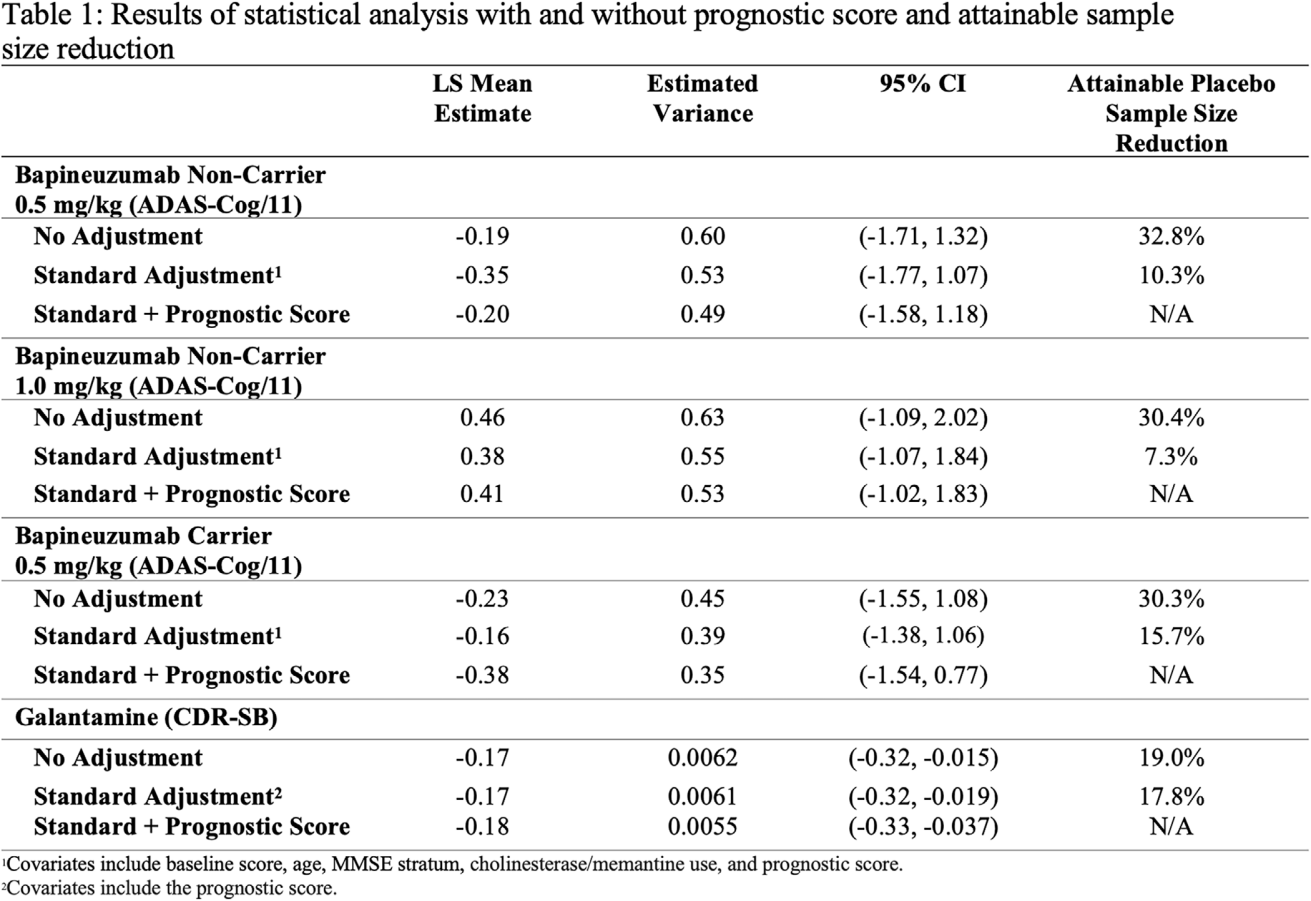# Accelerating randomized clinical trials in Alzheimer’s Disease using generative machine learning model forecasts of progression

**DOI:** 10.1002/alz.086486

**Published:** 2025-01-09

**Authors:** Amin Yakubu, Jennifer Bogert, Run Zhuang, Gayle Wittenberg, Christine Pozniak, Yi Zhang, Coco Kusiak, Michelle Turner, Bryan Hansen, Jonathan R. Walsh

**Affiliations:** ^1^ Johnson & Johnson Innovative Medicine, Cambridge, MA USA; ^2^ Janssen Research & Development, LLC, Raritan, NJ USA; ^3^ Unlearn.AI, San Francisco, CA USA; ^4^ Johnson & Johnson Innovative Medicine, Titusville, NJ USA; ^5^ Johnson & Johnson Innovative Medicine, San Francisco, CA USA; ^6^ Johnson & Johnson Innovative Medicine, New Brunswick, NJ USA; ^7^ Johnson & Johnson Innovative Medicine, Spring House, PA USA

## Abstract

**Background:**

Pivotal Alzheimer’s Disease (AD) trials typically require thousands of participants, resulting in long enrollment timelines and substantial costs. We leverage deep learning predictive models to create prognostic scores (forecasted control outcome) of trial participants and in combination with a linear statistical model to increase statistical power in randomized clinical trials (RCT). This is a straightforward extension of the traditional RCT analysis, allowing for ease of use in any clinical program. We demonstrate the application of these methods retrospectively on 3 pivotal Phase III clinical trials in mild‐to‐moderate AD (NCT00236431, NCT00574132, and NCT00575055).

**Method:**

A probabilistic deep learning model was trained on the trajectories of nearly 7000 participants who had varying degrees of cognitive impairment, ranging from mild cognitive impairment (MCI) to moderate AD. These trajectories were collected observational studies and the control arms of RCTs. This trained model was used to forecast the control outcomes of participants in the three trials retrospectively, by entering their individual trial baseline data. The resultant forecasts are known as prognostic scores and represent comprehensive predictions across a broad range of AD outcomes.

We evaluated the potential reduction in estimated variance and how this could translate to required sample size by incorporating the prognostic score as a covariate in the primary linear statistical model of each study, analyzing the 11‐component Alzheimer’s Disease Assessment Scale‐Cognitive Subscale (ADAS‐Cog11) and the Clinical Dementia Rating Sum‐of‐Boxes (CDR‐SB) endpoints as applicable.

**Result:**

Prognostic scores have the potential to decrease estimated variance between 5% to 10% and placebo arm sample size between 7% and 17% in the 3 studies when comparing standard + prognostic score vs. standard adjustment.

**Conclusion:**

Prognostic scores have the potential to increase the statistical power in clinical trials; this would enable a reduced number of subjects required to detect a significant treatment effect. Potential sample size reduction during trial planning must be carefully estimated using independent validation studies to reduce the risk of under‐powering the trial.